# ON THE FRONTLINES: TRAINING A DEMENTIA CAREGIVING WORKFORCE IN CALIFORNIA

**DOI:** 10.1093/geroni/igad104.3507

**Published:** 2023-12-21

**Authors:** Brittney Pond, Corinne Eldridge, Mel Neri, Matthew Beld, Leslie Ross, Suzanna Martinez, Brooke Hollister, Jarmin Yeh

**Affiliations:** University of California, San Francisco, San Francisco, California, United States; Center for Caregiver Advancement, Los Angeles, California, United States; University of California, San Francisco, San Francisco, California, United States; University of California, San Francisco, San Francisco, California, United States; University of California, San Francisco, San Francisco, California, United States; University of California, San Francisco, San Francisco, California, United States; Universitetet i Agder, Agder, Aust-Agder, Norway; University of California, San Francisco, San Francisco, California, United States

## Abstract

Direct care workers who provide in-home care – such as caregivers hired by consumers of California’s Medicaid-funded In-Home Supportive Services (IHSS) program – are well positioned to observe changes in their consumer’s cognition, health, or behaviors. This proximity to consumers better allows for reporting to healthcare partners, which may reduce risk of emergency department visits, hospitalizations, and other adverse outcomes that are disproportionately high among people living with Alzheimer’s disease and related dementias (ADRD). Despite their vital role in supporting people living with ADRD, IHSS caregivers receive minimal formal training for their unique position. To address this training gap, the IHSS+ ADRD Training Project is a five-year effort funded by the California Department of Public Health to train 720 IHSS caregivers in Alameda and Los Angeles counties, with classes in English, Spanish, Cantonese, and Mandarin. To date, 582 IHSS caregivers have been trained. Using a pre-posttest design, our preliminary findings indicate statistically significant increases in dementia knowledge and self-efficacy that could bolster caregiver’s skills. These results also reveal that IHSS caregivers value training opportunities to better support their consumers. Correlations between caregiver training outcomes and healthcare utilization patterns of their consumers were further examined by evaluating administrative data before and after the training. The results need to be further understood in the context of COVID-19 and the ADRD illness trajectory. Implications include expanding long-term funding for multi-week training programs, which aligns with the California Master Plan for Aging’s goal to support and create one million high-quality caregiving jobs by 2030.

